# Is intraoperative embolization with n-butyl cyanoacrylate an alternative option in carotid body tumors surgery? A case report

**DOI:** 10.1016/j.ijscr.2023.108636

**Published:** 2023-08-09

**Authors:** Nurkay Katrancioglu, Faruk Serhatlioglu, Ozgur Katrancioglu

**Affiliations:** a Department of Cardiovascular Surgery, Malatya Turgut Ozal University, School of Medicine, Malatya, Turkey; b Department of Cardiovascular Surgery, Niğde Ömerhalis Demir University, School of Medicine, Niğde, Turkey; c Department of Thoracic Surgery, Malatya Turgut Ozal University, School of Medicine, Malatya, Turkey

**Keywords:** Carotid body tumor, Intraoperative embolization, n-butyl cyanoacrylate, Glomus tumor

## Abstract

**Introduction and importance:**

The risk of intraoperative bleeding is relatively considerable because carotid body tumors (CBT) have rich vascular structures. Aim is to reduce intraoperative bleeding with preoperative embolization. We present a unique technique for the successful surgical removal of a challenging CBT using intraoperative direct percutaneous intratumoral n-butyl cyanoacrylate (n-BCA) embolization in a patient whose preoperative embolization failed and the operation could not be continued due to intraoperative bleeding.

**Clinical presentation:**

A 67-year-old female patient presented with 7 cm Shamblin class 3 CBT on her right neck. Due to the failure of the preoperative embolization, bleeding developed during the operation. In the case of Shamblin class 3 CBT, the primary concern was not the volume of bleeding, but the difficulty in seeing the dissection line due to hemorrhage. Intraoperative n-BCA straight embolization totally controlled the bleeding. The CBT was then readily removed.

**Clinical discussion:**

Effective management of intraoperative hemorrhage is essential to ensure successful progression of surgical procedures of CBT. Hemorrhage causes complete disappearance of the dissection line, which is already difficult to detect due to adventitia invasion. It is clear that another method is needed when preoperative embolization or covered stenting fails. n-BCA has been used in the endovenous treatment of varicose veins for a long time, but to the best of our knowledge, there is no other case of its use in intraoperative CBT embolization.

**Conclusion:**

Direct intraoperative embolization with n-BCA may be an alternative when other techniques are insufficient.

## Highlights

• The management of intraoperative bleeding in CBT surgery is crucial.
• Preoperative embolization is a standard method to reduce intraoperative bleeding.
• Non-catheterizable feeding arteries are problem for intraoperative embolization.
• Failed preoperative embolization requires alternative approaches.
• Intraoperative embolization with n-butyl cyanoacrylate may be an alternative.

## 1 Introduction

Carotid body tumors (CBT) are usually benign and highly vascular tumors. Its incidence is about 1 in 30,000 [1]. Surgical resection is the most effective treatment method for resectable CBTs. The highly vascular nature of CBTs sometimes makes surgical resection difficult. Despite advances in surgical technique, intraoperative bleeding in surgical resection of CBT is still a challenging problem. Traditionally, preoperative embolization aims to occlude feeding artery of the CBT to reduce intraoperative bleeding. However, in certain instances of Shamblin Class 3 cases, the effectiveness of preoperative embolization may be limited due to the presence of multiple small feeder branches originating from the internal carotid artery (ICA) or external carotid artery (ECA), which cannot be catheterized directly. As a result, there is a chance that the surgical procedure will result in uncontrolled bleeding. In this case report, we describe the successful surgical removal of a Shamblin Class 3 CBT case that underwent intraoperative embolization with direct percutaneous intratumoral n-butyl cyanoacrylate (n-BCA) injection using a unique technique in a patient who was unable to undergo preoperative embolization and experienced uncontrolled intraoperative bleeding. The article was reported in accordance with SCARE criteria [2].

## 2 Case report

**Fig. 1:**
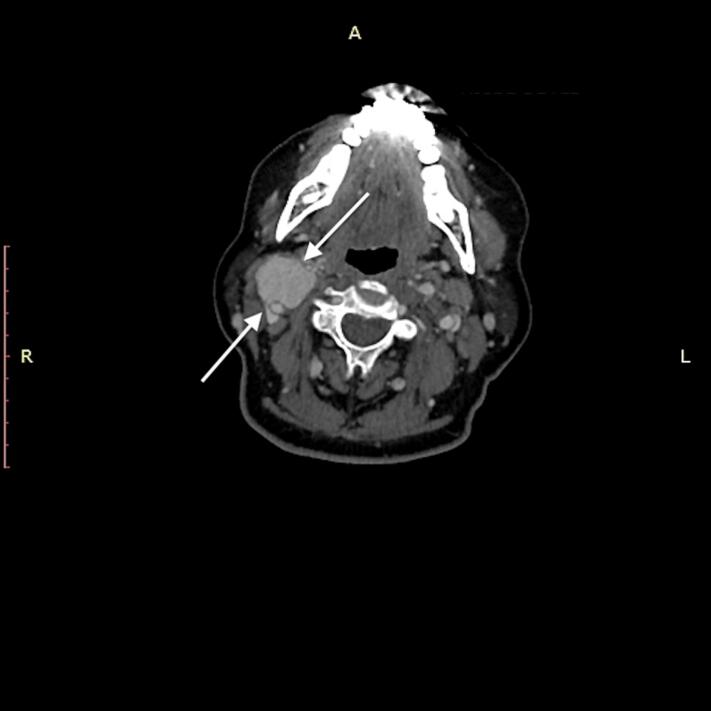
Computed tomography image of the carotid body tumor.

A 67-year-old woman presented with a complaint of a painless, pulsatile mass in the right side of her neck. The mass has been progressively growing for the past 9 months. Other than a headache, she did not describe any additional symptoms. Her drug history, psychological history, and family history, including relevant genetic information, were all negative. A 7 cm pulsatile and painless mass was found in the right anterolateral neck during physical examination. Cranial nerve examination was normal, and routine laboratory blood tests were within normal limits. Doppler ultrasonography revealed a 51 × 44 mm hyper vascular mass located at the common carotid artery (CCA) bifurcation, which displaced the ICA and ECA. Computed tomography described a Shamblin Class 3 CBT located at the carotid bifurcation and extending distally to the level of the skull base, making surgical reconstruction difficult (Fig. 1). Preoperative embolization was planned, however, because of the presence of minor feeding branches from the ICA and ECA, preoperative embolization was not as successful as planned. After obtaining informed consent from the patient, the patient underwent surgery.

### 2.1 Operative technique

**Fig. 2:**
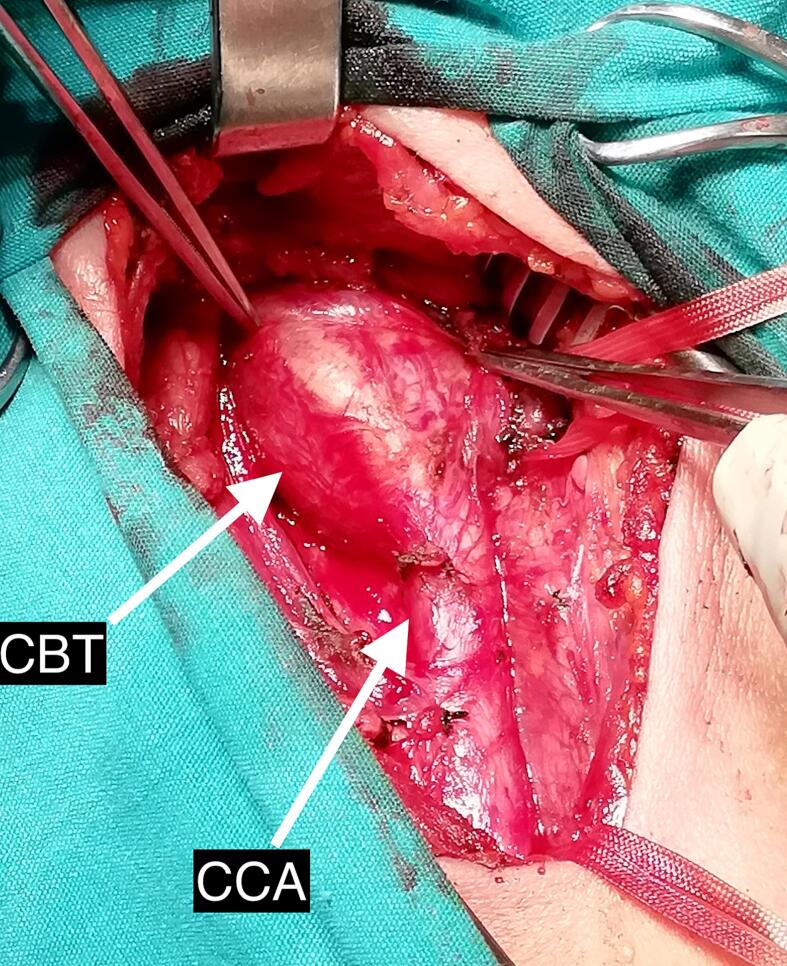
Intraoperative image of carotid body tumor.

The patient was operated by an experienced cardiovascular surgeon. An anterior skin incision was made along the sternocleidomastoid muscle border under general anesthesia. The CCA and its surrounding tissue were mobilized, and vessel loops were used to encircle the CCA at the intended site, as well as the ICA and ECA, just below the mandible, within the tumor-free zone (Fig. 2). During the sub-adventitial dissection of the CBT profuse bleeding occurred. Despite efforts to control the bleeding by isolating and tying specific vascular supplies of the CBT, hemorrhage persisted. As a result of the uncontrolled bleeding, further dissection and resection of the CBT became impossible. To address this challenge, a decision was made to perform intraoperative embolization of the CBT using n-BCA. To prevent distal embolization during the procedure CCA was clamped, 0.5 ml of n-BCA (VariClose, Biolas, Ankara, Turkey) was injected directly into the avascular tumor site in the body of the CBT in the middle of the ICA and ECA. After a one-minute waiting period, the clamp was released.

**Fig. 3:**
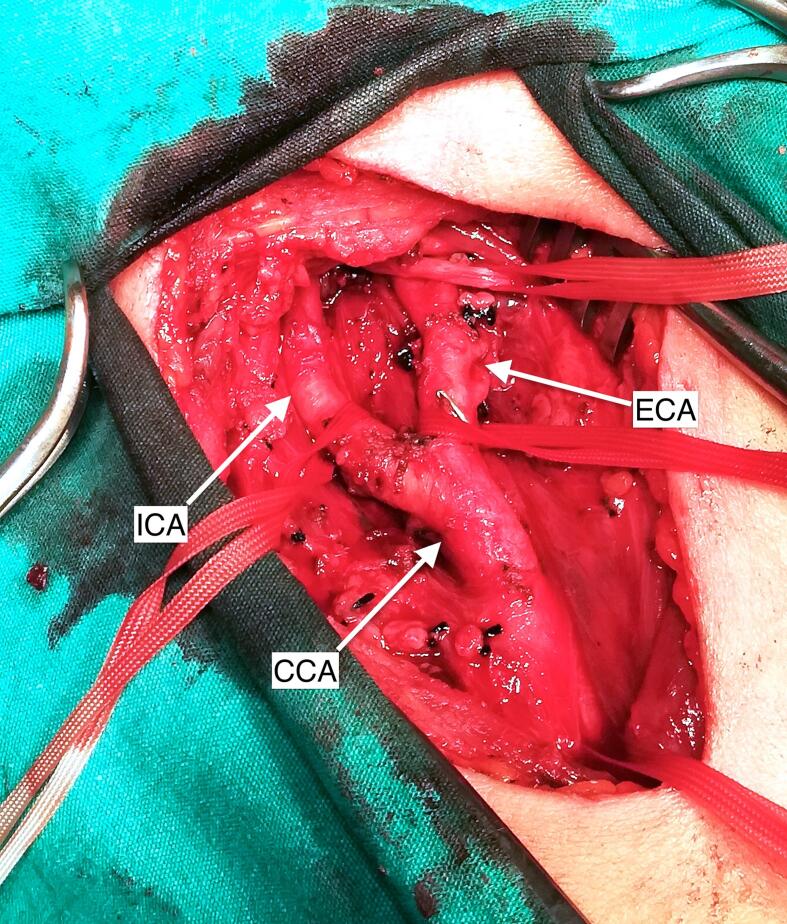
Surgical view of internal, external and common carotid artery after carotid body tumor excision.

**Fig. 4:**
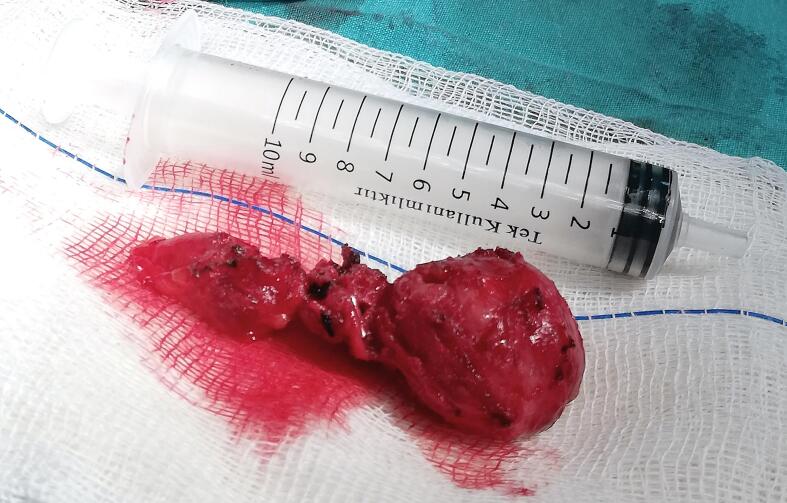
Surgically excised carotid body tumor.

Following the intraoperative embolization of the CBT, bleeding stopped immediately, allowing surgical dissection and total tumor removal to resume. Despite the CBT being classed as Shamblin Class 3 and invading the ICA and ECA walls at the carotid bifurcation, the presence of a defined dissection line allowed for its easy removal (Fig. 3, Fig. 4).

After the intraoperative embolization, the intraoperative total blood loss was less than 50 cc. Postoperative period was uneventful, with no observed deficits in lower cranial nerves or any vascular complications. The patient did not report any complaints that differed from typical carotid surgery patients. Histopathological analysis confirmed the diagnosis of a CBT. The patient was discharged home on the fourth postoperative day. The patient was followed up for two years postoperatively. During the follow-up of the patient, there was no recurrence or a situation requiring new intervention.

## 3 Discussion

The rich blood supply of the CBT sometimes makes its resection extremely difficult, especially in Shamblin Class 3 cases. Intraoperative hemorrhage due to the rich blood supply not only can cause blood to lose but also increases incidence of surgical complications such as vascular and cranial nerve injury [3].

According to a meta-analysis, patients who underwent embolization before CBT resection experienced significantly reduced blood loss and shorter operation durations [4]. Embolization of CBT's main arterial supply within preoperative 48 h can reduce CBT size and incidence of surgical complication related with removal of CBT [5,6]. However, the efficacy of preoperative embolization may be limited in the presence of extremely small feeder branches from ICA or ECA. We planned to perform preoperative embolization in our case, but embolization failed due to the presence of multiple small feeder branches originating from the ICA and ECA, which cannot be catheterized directly. The current literature does not offer adequate information to predict the presence of multiple small, non-catheterizable feeding arteries before the procedure. However, it is worth speculating that such occurrences might be more prevalent in Shamblin Class 3 cases. Preoperative covered stent placement in the ECA to close the branches feeding the CBT may also be considered as an alternative technique [7]. However, covered carotid stents have some potential risks, such as in-stent stenosis, high cost, and the lack of long-term patency data [8]. Therefore, we could not use covered stent in our case.

Effective management of intraoperative bleeding is essential to ensure successful progress of surgical procedures. Uncontrolled bleeding during surgery can interrupt the surgical process and necessitate immediate measures to manage it. Due to uncontrollable bleeding, the surgical procedure became nearly impossible. In this situation, preoperative embolization seemed the only viable solution. Preoperative direct intratumoral embolization of CBT has been reported in several articles in the literature, but as far as we can see, the use of intraoperatively has not been reported yet [9,10]. In our clinic, n-BCA is frequently used in the endovenous treatment of varicose veins. We preferred to use n-BCA, both inspired by the studies in the literature and as an easily accessible embolization agent in our clinic. We were encouraged by this procedure when the hemorrhage stopped right away during intraoperative embolization. Although we did this procedure because we couldn't find a better solution, it raises the question of whether it may be considered at the start of the operation without bleeding in large size Shamblin class 3 CBT instances when other methods cannot be utilized for various reasons.

Potential adverse effects of direct embolization have been reported as embolization agent migration, chemical toxicity, or cranial nerve injury [9,10]. Cranial migration of embolization agent is one of the most frightening complications [10]. It can be prevented by using more than sufficient amount of embolizing agent and stopping the active blood flow during the procedure [9]. Therefore, direct CBT embolization was performed using 0.5 ml n-BCA under the CCA clamp. Although not seen in our case, Krishnamoorthy et al. reported that CBT delayed stroke after direct embolization [11]. We think that such complications may be related to the amount of embolizing agent. To prevent cranial nerve injury, another important complication, the locations of adjacent cranial nerves were determined before the embolization, and contact with the embolic agent was avoided. There were no signs of distal migration or cranial nerve damage either in the early or late postoperative period.

## 4 Conclusion

CBT surgeries are associated with a substantial risk of bleeding, particularly in Shamblin class 3 cases. Preoperative embolization or covered stenting is an option; however, it may not be applicable in all cases. Although one case is not sufficient to reach a definitive conclusion, intraoperative embolization with n-butyl cyanoacrylate seems to be an alternative option for carotid body tumors surgery. Further studies are needed to substantiate our findings.

## Patient consent

Yes.

## Provenance and peer review

Not commissioned, externally peer-reviewed.

## Ethical approval

N/A.

## Funding

None.

## Authors contribution

Nurkay Katrancioglu: Idea/Concept, Writing the Article.

Faruk Serhatlioglu: Analysis and interpretation, Materials.

Ozgur Katrancioglu: Literature Review, Critical Review.

## Guarantor

Prof. Dr. Nurkay Katrancioglu.

## Research registration number

None.

## Conflict of interest statement

None.

## Data Availability

Yes.
